# Oral Frailty: Current Concepts, Assessment, Systemic Implications, and Future Perspectives

**DOI:** 10.3390/geriatrics11050137

**Published:** 2026-10-01

**Authors:** Hiroshi Kusunoki, Ken Shinmura, Hideo Shimizu

**Affiliations:** 1 Department of Internal Medicine, Osaka Dental University, 8-1 Kuzuhahanazonocho, Hirakata 573-1121, Osaka, Japan; taropapa@mx6.nisiq.net; 2 Department of General Internal Medicine, School of Medicine, Hyogo Medical University, Nishinomiya 663-8501, Hyogo, Japan; ke-shimmura@hyo-med.ac.jp

**Keywords:** oral frailty, sarcopenia, screening, cystatin C, medical–dental collaboration

## Abstract

Oral frailty is an emerging geriatric syndrome characterized by subtle and multifactorial declines in oral function that may precede clinically apparent oral dysfunction. Increasing evidence suggests that oral frailty is closely associated with physical frailty, sarcopenia, malnutrition, and other indicators of systemic vulnerability, highlighting its potential role in the early detection of functional decline. However, practical approaches to early detection and intervention remain challenging because conventional assessments of oral function often require specialized equipment and professional expertise. This review summarizes recent advances in the assessment, clinical significance, and management of oral frailty, with particular emphasis on practical screening and its implications for early intervention. We discuss the utility and limitations of questionnaire-based screening tools, including the Oral Frailty Index-8 (OFI-8) and Oral frailty five-item checklist (OF-5), which enable simple identification of individuals at increased risk in community and clinical settings. We also introduce findings from the FESTA study indicating that cystatin C-related indices, including the creatinine-to-cystatin C ratio and the eGFRcys/eGFRcre ratio, may reflect skeletal muscle mass and are associated with oral functional decline, particularly reduced tongue pressure. Comparative and longitudinal evidence further suggests an association between oral frailty and subsequent physical frailty, while OF-5 may help identify individuals at risk of functional decline. Because oral and physical frailty may influence each other bidirectionally, management should extend beyond screening to include appropriate oral care, nutritional support, physical exercise, and medical evaluation. Integrating simple oral frailty screening with objective systemic indicators may facilitate earlier identification of vulnerable individuals and strengthen medical–dental collaboration aimed at preventing frailty progression.

## 1. Introduction

In recent years, accumulating evidence has demonstrated that even subtle declines in oral function are closely associated with physical frailty, sarcopenia, functional disability, and mortality among older adults [1,2,3]. Oral frailty has therefore gained recognition as an important geriatric syndrome representing an early and potentially reversible stage between healthy oral status and clinically apparent oral functional decline. It is characterized by the accumulation of mild, multifactorial impairments across oral domains, including tooth loss, mastication, swallowing, speech, and salivary function. Similar to physical frailty, this transitional state may provide an opportunity for early identification and intervention to prevent further functional deterioration.

In 2024, a joint statement from the Japan Geriatrics Society, the Japanese Society of Gerodontology, and the Japanese Association on Sarcopenia and Frailty formally defined oral frailty as an intermediate condition between healthy oral status and oral functional decline [4]. The statement emphasized that accumulating subtle impairments in eating, communication, and other oral functions may increase the risk of subsequent functional decline while remaining potentially modifiable. This concept positions oral frailty not simply as a dental condition but as a potential target for maintaining functional independence and promoting healthy aging.

The clinical significance of oral frailty extends beyond oral health. It has been associated with physical frailty, sarcopenia, malnutrition, cognitive impairment, and social vulnerability [5,6,7,8]. The Kashiwa Study showed that older adults with both oral and physical frailty had the highest cumulative risks of disability and mortality [2], while longitudinal studies suggest that oral functional decline may precede physical frailty and locomotor dysfunction [2,9]. Associations have also been reported with cardiovascular disease, multimorbidity, psychological well-being, and cognitive decline [10,11]. Thus, oral frailty may reflect broader biological, psychological, and social vulnerability rather than isolated deterioration of oral function. Nevertheless, the mechanisms and directionality of these associations remain incompletely understood, and cross-sectional associations should be distinguished from longitudinal evidence of temporal or predictive relationships.

Despite growing evidence linking oral frailty to adverse health outcomes, the optimal strategies for screening and intervention remain under investigation. Therefore, effective management requires a multidisciplinary approach integrating comprehensive dental care, oral functional training, nutritional support, physical exercise, and interventions that promote social participation [12,13,14,15].

Questionnaire-based screening tools, particularly the Oral Frailty Index-8 (OFI-8) and Oral frailty five-item checklist (OF-5), provide practical approaches for identifying individuals at risk, but they differ in constructs, scoring systems, and intended applications, and their validity and clinical utility across populations remain under investigation [16]. Conversely, conventional objective assessments of oral function may require specialized equipment and professional expertise, limiting their use in primary care and community settings. Objective systemic biomarkers that can complement questionnaire-based screening have therefore attracted increasing interest. Moreover, compared with the substantial evidence linking oral frailty to systemic disease, functional disability, and mortality, evidence supporting effective prevention and intervention remains relatively limited.

Against this background, this review examines the practical clinical continuum from recognition and screening to objective assessment, interpretation of systemic vulnerability, and subsequent prevention and management. We summarize current conceptual, epidemiological, and pathophysiological evidence; critically review screening and assessment approaches, with particular emphasis on the OFI-8 and OF-5; and discuss emerging objective biomarkers that may complement questionnaire-based screening. We further examine current evidence on prevention and intervention and consider the integration of oral frailty assessment into multidisciplinary medical–dental care. Through this integrated perspective, we aim to distinguish established evidence from emerging hypotheses and identify practical directions for the early detection and management of oral frailty.

## 2. Assessment, Screening, and Management Strategies for Oral Frailty

Recent systematic reviews and meta-analyses have estimated the pooled prevalence of oral frailty among older adults at approximately 28–34%, although estimates vary substantially across studies, with very high statistical heterogeneity (I^2^ ≈ 99%). Similar estimates of approximately 32–34% have been reported among community-dwelling older adults [17,18,19,20]. However, prevalence varies considerably according to the assessment instrument; one meta-analysis, for example, reported pooled prevalences of 46% for OFI-8, 37% for OF-5, and 18% for OF-6 [20]. These estimates should therefore be interpreted as descriptive summaries of heterogeneous populations and operational definitions rather than definitive population prevalence. Prevalence estimates derived from different instruments should not be directly compared without considering differences in item composition, scoring systems, cutoff values, and target populations.

Comprehensive oral examinations and objective assessments provide valuable clinical information but may be difficult to implement in community settings, primary care, and large-scale epidemiological studies. Measures such as tongue pressure, occlusal force, masticatory performance, swallowing function, salivary secretion, and oral diadochokinesis (ODK) often require specialized equipment, trained personnel, or relatively complex testing [3,4,21].

These practical limitations have increased interest in simpler questionnaire-based instruments, particularly the OFI-8 and OF-5, for initial identification of individuals at risk of oral frailty.

Oral frailty should, however, be distinguished from oral hypofunction. Oral hypofunction is an objective clinical framework developed in Japan and is diagnosed when impairment is present in at least three of seven domains: poor oral hygiene, oral dryness, reduced occlusal force, decreased tongue–lip motor function, low tongue pressure, impaired masticatory function, and impaired swallowing function [22] (Table 1).

In contrast, oral frailty represents an earlier, intermediate, and potentially reversible state between healthy oral status and clinically apparent functional decline, characterized by the accumulation of mild impairments across multiple domains, including tooth loss, chewing, eating, swallowing, and communication [1]. Although the two concepts overlap, their precise conceptual and temporal relationship has not been fully established. Accordingly, oral hypofunction is considered in this review as a complementary framework for characterizing established oral functional impairment rather than an alternative definition of oral frailty. Questionnaire-based oral frailty screening may identify individuals with early multidimensional vulnerability, whereas objective assessment of oral hypofunction can provide a more detailed characterization of specific functional deficits.

Questionnaire-based and objective assessments should therefore be regarded as complementary rather than competing approaches. The OFI-8 and OF-5 are simple and readily applicable for community-based screening but may not fully capture objectively measurable impairment. Conversely, objective measures provide more direct assessment of specific oral functions but are less feasible for large-scale screening. This distinction is illustrated by the articulatory oral motor function item of the OF-5, which relies on self-reported difficulty with clear pronunciation, compared with ODK, which objectively measures repetitive articulation of syllables such as /pa/, /ta/, and /ka/ and is incorporated into the oral hypofunction framework [2]. Although the self-reported item showed good agreement with ODK (κ = 0.71), its sensitivity was relatively low (42.1%) despite high specificity (83.1%), suggesting that objective ODK may detect some impairments not identified by self-report [23]. However, whether adding ODK to the OF-5 improves the overall diagnostic or predictive accuracy of oral frailty screening remains unknown. Given the predictive validity of the OF-5 for physical frailty, long-term care needs, and mortality, and its moderate discrimination for objectively diagnosed oral hypofunction (AUC = 0.781), OF-5 may be suitable as a simple first-line screening tool in community settings, while objective measures such as ODK can be added for more detailed assessment [2,24].

A practical clinical pathway may therefore begin with brief questionnaire-based screening, followed by targeted objective oral assessment and evaluation of nutritional, physical, and systemic vulnerability in individuals who screen positive. This two-stage approach may combine the feasibility of population-level screening with the greater specificity of objective assessment and facilitate subsequent individualized, multidisciplinary intervention. Further prospective studies are needed to determine whether integrating objective oral measures and readily available systemic biomarkers with simple screening instruments improves early detection, risk stratification, and clinical outcomes.

## 3. Comparison of Oral Frailty Screening Tools and Their Clinical Utility

Given the high prevalence and clinical implications of oral frailty, simple and practical screening approaches are increasingly important. The OFI-8 is one of the most widely used instruments in Japan. Developed as a patient-reported risk-assessment tool, it comprises eight items covering dental attendance, dentition, chewing ability, dietary habits, oral functional problems, and oral health awareness, with a maximum score of 11. A score of ≥4 was originally reported to discriminate oral frailty with good accuracy (AUC 0.88, 95% CI 0.86–0.90; sensitivity and specificity, 80%), and higher scores have been associated with subsequent oral frailty and functional disability [16]. The OFI-8 may also capture broader health-related behaviours, as greater oral frailty awareness has been associated with exercise, balanced nutrition, and oral health-promoting behaviours [25].

However, the OFI-8 should primarily be regarded as a tool for risk screening rather than as a diagnostic instrument. It was originally developed in Japanese populations, and its validity and generalizability across different populations and cultural settings remain uncertain. A 2022 scoping review highlighted limited evidence regarding the validity and predictive performance of oral frailty assessment tools, including the OFI-8 [21]. More recent validation studies have raised further concerns. In 227 community-dwelling adults in Singapore, the OFI-8 showed moderate internal consistency (Cronbach’s α = 0.61) and test–retest reliability (intra-class correlation coefficient = 0.62), but limited structural validity (comparative fit index = 0.77); associations with objective oral measures were modest, and the conventional cutoff of ≥4 did not discriminate participants according to functional tooth number [26].

The OF-5 was developed as a simpler instrument for rapid screening. It consists of five self-reported items addressing tooth loss, chewing difficulty, swallowing difficulty, reduced oral motor function, and dry mouth, with oral frailty generally defined as two or more positive items [2]. The OF-5 has demonstrated associations with physical frailty, disability, and mortality, as well as with objectively diagnosed oral hypofunction [24]. However, it does not adequately assess oral hygiene-related conditions, such as periodontal disease and plaque accumulation, and therefore should complement rather than replace comprehensive dental examination.

**Figure 1:**
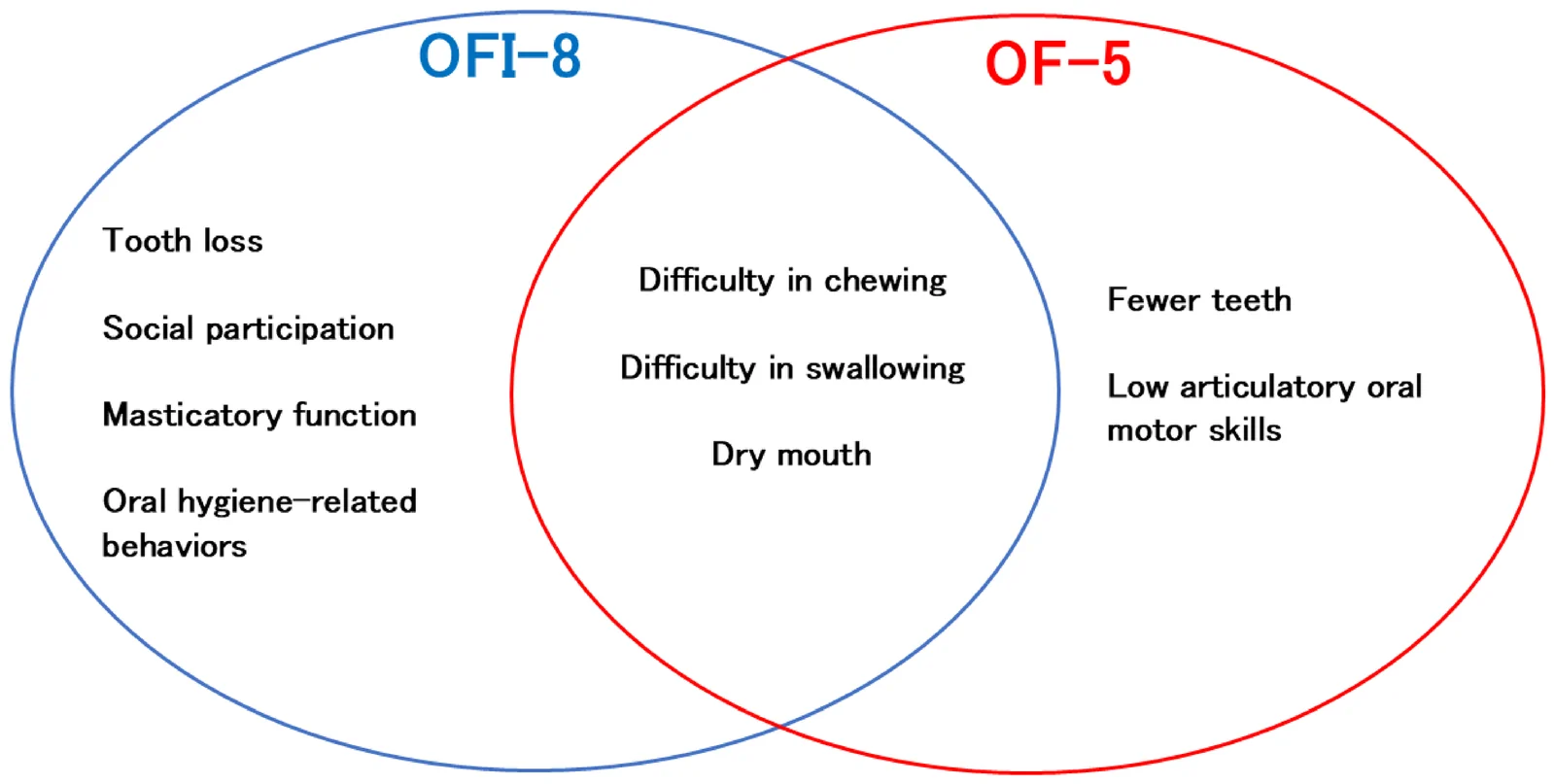
Overlap between the OFI-8 and OF-5. Created by the authors based on References [4,16].

Despite differences in structure and scope, the OFI-8 and OF-5 share three core components—difficulty in chewing, swallowing, and dry mouth—which may partly explain their substantial concordance (Figure 1). A strong correlation between the two instruments has previously been reported [26] (r = 0.72, *p* < 0.001).

We developed a questionnaire enabling simultaneous administration of the OFI-8 and OF-5 (Table 2) and conducted a cross-sectional study of 270 Japanese adults aged ≥65 years attending a general internal medicine outpatient clinic between February and September 2024 [27].

**Figure 2:**
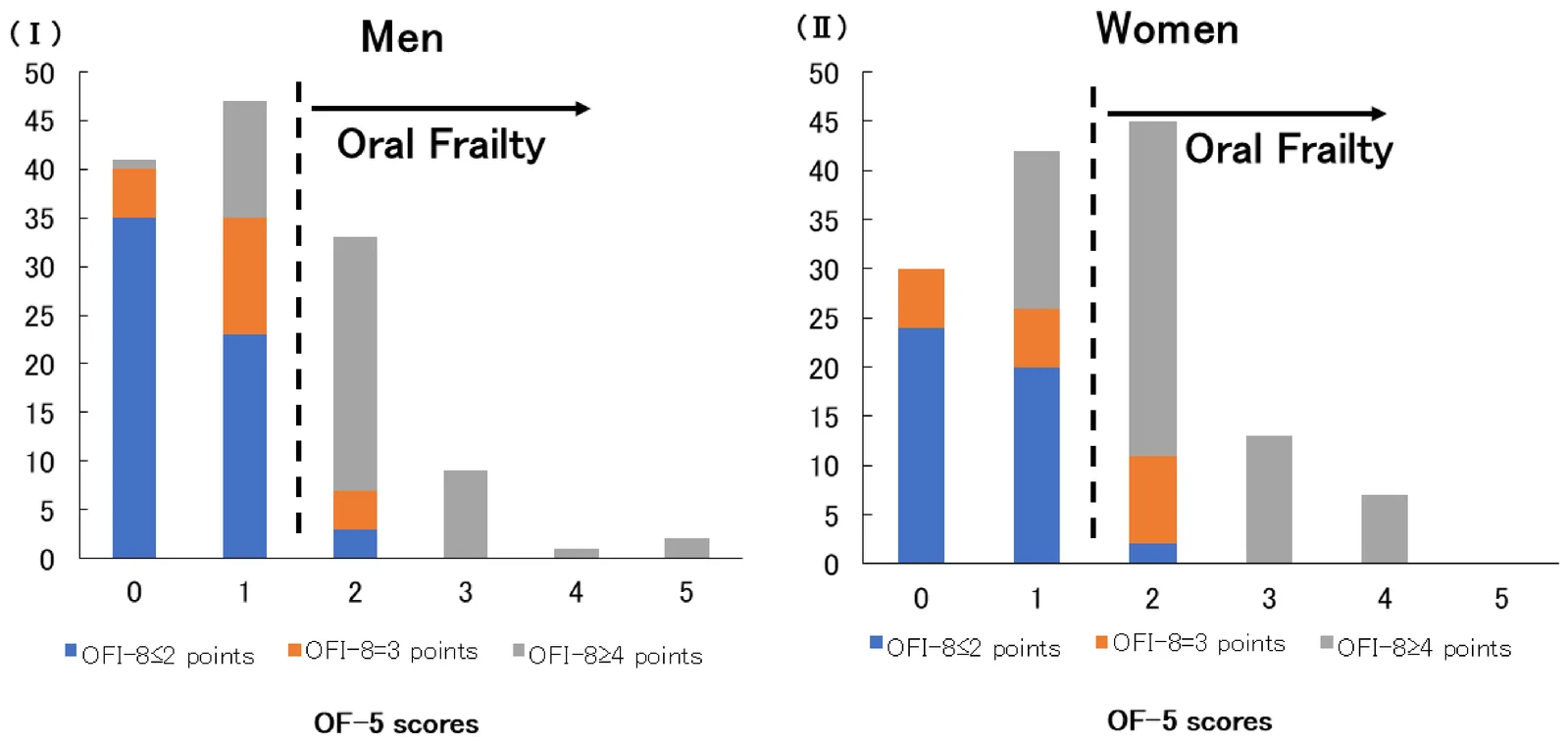
Risk stratification of OFI-8 according to OF-5 scores in general internal medicine outpatients. Reproduced from Reference [27].

**Figure 3:**
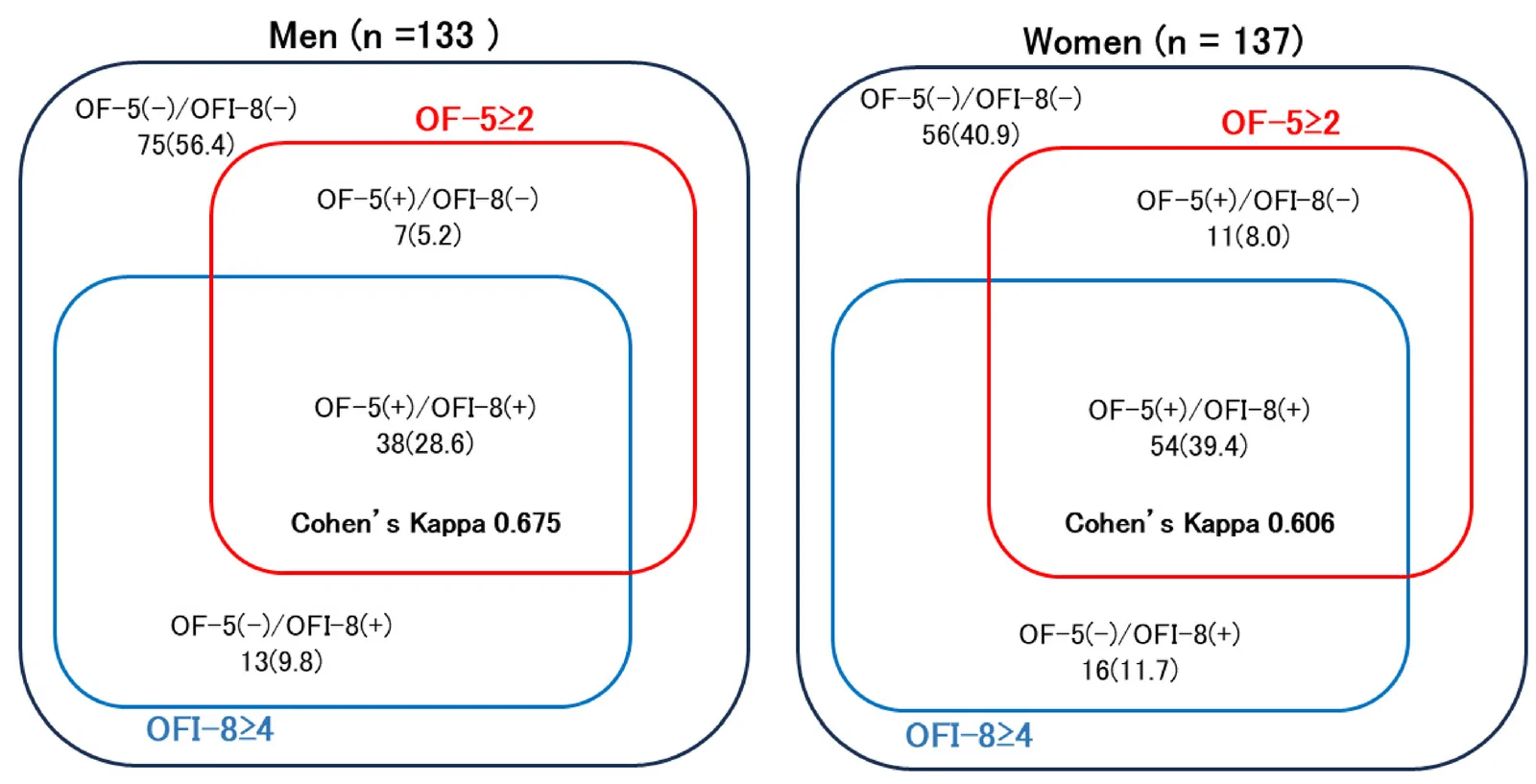
The proportions of individuals with an OF-5 score ≥ 2 (defined as OF-5 (+)) and an OFI-8 score ≥ 4 (defined as OFI-8 (+)) within the overall sample in men (A) and women (B). Notes: x (y), number of patients (%). Reproduced from Reference [27].

All participants completed both questionnaires and underwent physical measurements, grip-strength assessment, and blood testing. Oral frailty was defined as an OF-5 score of ≥2, whereas an OFI-8 score of ≥4 indicated high risk. Overall, 110 participants (40.7%) met the OF-5 criterion and 121 (44.8%) met the OFI-8 criterion. Notably, all participants with OF-5 scores ≥ 3 were classified as high risk by the OFI-8 (Figure 2). Among those identified by the OF-5, approximately 83–84% were also classified as high risk by the OFI-8 (Figure 3). Agreement was substantial in both men and women (Cohen’s κ = 0.675 and 0.606, respectively), and ROC analyses identified an OFI-8 cutoff of 4 in both sexes, consistent with the conventional threshold [27]. Participants classified as high risk by the OFI-8 also had lower handgrip strength, while men additionally had lower erythrocyte counts and serum albumin levels.

These findings support complementary clinical roles for the two instruments. The OF-5 may be particularly suitable for rapid community or population-level screening because of its brevity, simple scoring, and ease of administration without specialized equipment. The OFI-8, by incorporating dental care, dietary habits, oral health behaviours, and awareness, may provide broader risk stratification. Nevertheless, neither instrument should be considered a diagnostic gold standard or a substitute for comprehensive oral functional assessment and dental examination. The cross-sectional design and selected outpatient population of our comparative study also limit generalizability.

Accordingly, a positive screening result should prompt further multidimensional evaluation, including objective oral functional assessment, dental examination, and assessment of nutritional, physical, and systemic vulnerability. In this context, several multidimensional patient-reported approaches have recently been explored. The 24-Item Oral Frailty Scale (OF-24) was developed through a three-round international e-Delphi process involving 25 experts in geriatric dentistry, oral rehabilitation, geriatric medicine, rehabilitation, and population oral health [28]. The scale was designed to capture eight components across four domains—mastication, swallowing, oral motor skills, and salivation—based on a recently established operational definition of oral frailty. Although OF-24 provides a potentially comprehensive framework for patient-reported assessment, further evaluation across diverse populations and languages is required before its widespread adoption.

More recently, the Oral Frailty 20-item Checklist (OF-20) was developed by the Sakai District Dental Association of Fukui Prefecture [29,30] and has been explored as a multidimensional profiling instrument that may complement, rather than replace, established oral frailty screening tools. In a cross-sectional study of 297 older adults attending internal medicine outpatient clinics in Japan, 39.4% met the conventional OF-5 criterion for oral frailty, yet OF-20 domain scores varied substantially among these individuals, suggesting heterogeneous patterns of oral and systemic vulnerability that may not be captured by a binary OF-5 classification. Associations of specific OF-20 domains with age, body weight, grip strength, hemoglobin levels, renal function, and selected comorbidities further suggest that oral frailty may encompass distinct multidimensional phenotypes [31]. Future prospective studies should determine whether combining questionnaire-based screening with multidimensional profiling, objective oral functional measures, and readily available systemic biomarkers can improve early detection, risk stratification, and prediction of clinically relevant outcomes.

## 4. Assessment of Muscle Mass and Oral Function Using Cystatin C-Related Indices

Although questionnaire-based screening tools are practical and widely used, objective biomarkers that may reflect both systemic vulnerability and oral functional decline remain limited. In the FESTA study, a community-based cohort conducted by Hyogo Medical University in Tamba-Sasayama, we previously demonstrated that the creatinine-to-cystatin C ratio (Cr/CysC), which combines creatinine (Cr), a marker influenced by muscle mass, with cystatin C (CysC), which is relatively independent of muscle mass, is associated with skeletal muscle mass [32,33]. We further reported that cystatin C-based estimated glomerular filtration rate (eGFRcys) and the eGFRcys/eGFRcre ratio are associated with skeletal muscle mass and sarcopenia risk [34]. These findings are supported by studies in other populations, including community-dwelling older adults and individuals with chronic kidney disease, as well as by longitudinal evidence suggesting that certain creatinine- and cystatin C-derived indices may be associated with subsequent sarcopenia risk [35,36,37].

We subsequently investigated whether cystatin C-related indices might also be associated with oral function. Tongue pressure is an objective component of the oral hypofunction framework and has been linked to age, skeletal muscle mass, muscle strength, and physical performance. It has also been positively associated with eGFRcys but not eGFRcre [38]. These findings suggest that cystatin C-related measures may capture systemic characteristics relevant to both muscle status and age-related oral functional decline.

Consistent with this hypothesis, in a cross-sectional study of 251 older adults attending general internal medicine outpatient clinics, those with high OFI-8-defined oral frailty risk (OFI-8 ≥ 4) had lower Cr/CysC and eGFRcys/eGFRcre ratios than those at lower risk, together with lower handgrip strength in both sexes. Both indices were also significantly associated with oral frailty risk in receiver operating characteristic analyses [39]. Together, these findings support the potential utility of these indices as complementary indicators of systemic vulnerability associated with oral frailty.

The potential interpretation of these indices is biologically plausible. Because creatinine production is influenced by skeletal muscle mass whereas cystatin C is less dependent on muscle mass, Cr/CysC may provide information related to muscle-related vulnerability. Similarly, eGFRcys/eGFRcre may reflect discrepancies between cystatin C- and creatinine-based estimates of kidney function that are partly related to reduced creatinine generation in individuals with low muscle mass. However, these measures should not be interpreted as direct measures of muscle mass or oral function, and no biomarker has yet been established as a specific or superior biomarker of oral frailty.

Several limitations also need to be considered. Creatinine is influenced by muscle mass, physical activity, dietary protein intake, and medications that affect tubular creatinine secretion, whereas cystatin C, although less dependent on muscle mass, may be influenced by non-GFR determinants such as obesity, inflammation, glucocorticoid exposure, and thyroid dysfunction [40,41,42,43].

The diagnostic performance of Cr/CysC for sarcopenia has been variable across studies, with only moderate overall diagnostic accuracy, and eGFRcys/eGFRcre should be interpreted cautiously as a surrogate marker of muscle status because it reflects the combined influences of creatinine- and cystatin C-related determinants and renal function [34,44,45,46,47]. Associations with oral frailty may further be confounded by age, nutritional status, body composition, chronic disease, physical activity, inflammation, and renal dysfunction. Because most available evidence is observational or cross-sectional, it remains unclear whether changes in these indices precede oral functional deterioration, reflect broader systemic decline, or simply indicate greater multimorbidity (Table 3).

Accordingly, Cr/CysC and eGFRcys/eGFRcre should currently be regarded as hypothesis-generating or complementary markers of systemic vulnerability rather than validated diagnostic biomarkers of oral frailty. Further longitudinal studies are needed to determine whether they provide incremental predictive value beyond established measures of muscle mass, strength, and renal function, and whether their integration with OF-5 or OFI-8 screening can improve identification of individuals at risk of progression to oral hypofunction and physical frailty.

## 5. Longitudinal Findings and Predictive Factors in a Community-Based Cohort

Consistent findings were observed in the FESTA study. Participants with oral frailty (OF-5 score ≥ 2) were older and had significantly lower skeletal muscle mass, handgrip strength, knee extension strength, and gait speed, as well as poorer physical performance, reflected by longer Timed Up and Go and Five-Times Sit-to-Stand test times. They also had lower Cr/CysC and eGFRcys/eGFRcre ratios, while women additionally had lower serum albumin and hemoglobin levels [48]. Oral frailty was also strongly associated with physical frailty according to the J-CHS criteria, with lower prevalence of robust status and higher prevalence of prefrailty and frailty. These findings indicate that oral frailty is closely associated with multiple domains of physical vulnerability, including muscle mass, physical performance, nutritional status, and renal function.

The longitudinal analysis of the same cohort included 329 participants (105 men and 224 women) followed for 2–3 years. During follow-up, 27 of 105 participants (25.7%) in men and 47 of 224 participants (21.0%) in women experienced progression of physical frailty according to the J-CHS criteria. Although baseline body composition, handgrip strength, and gait speed did not differ significantly between participants whose frailty status subsequently deteriorated and those who remained stable, the total OF-5 score was significantly higher in the deterioration group. Logistic regression analyses identified sex-specific predictors, with decreased handgrip strength and tooth loss in men and impaired mastication and swallowing in women. Importantly, the OF-5 score remained an independent predictor of frailty progression in both sexes after adjustment for potential confounders [48]. These longitudinal findings suggest that oral frailty may precede measurable deterioration in physical frailty and may serve as an early marker of subsequent frailty progression.

In the same cohort of 413 community-dwelling older adults followed for a mean of 3.25 years, remaining teeth, occlusal force, and maximum tongue pressure declined, and greater declines in occlusal force were associated with higher odds of frailty worsening [49]. Together, these findings suggest that oral frailty and specific oral functional decline may occur in parallel with, or precede, broader frailty progression, although causality remains uncertain and the available evidence warrants cautious interpretation.

These observations also highlight the importance of viewing oral frailty screening as a starting point for prevention and intervention rather than an endpoint. Oral functional training may improve specific aspects of oral function, whereas multicomponent interventions incorporating physical activity and nutritional support may address multiple dimensions of oral frailty [15,50]. However, the optimal combination, intensity, and duration of these interventions, and whether they can prevent progression of oral or physical frailty and improve clinically meaningful outcomes, remain to be established.

## 6. Prevention and Intervention After Oral Frailty Screening

Screening for oral frailty should be regarded as the first step toward targeted prevention and intervention rather than an endpoint. Individuals who screen positive should undergo multidimensional assessment to identify predominant oral, nutritional, and physical deficits, followed by individualized intervention and periodic reassessment. Because oral frailty is multidimensional, management may require collaboration among dental professionals, physicians, dietitians, and rehabilitation professionals [4].

Interventions should be tailored to the identified deficits. Potentially modifiable oral problems, including poor oral hygiene, xerostomia, tooth loss, impaired mastication, swallowing difficulties, and reduced oral motor function, should be addressed through appropriate dental treatment, oral functional exercises, and, when indicated, nutritional support and physical exercise. Nutritional assessment and dietary counseling are particularly relevant in individuals with weight loss, poor appetite, reduced dietary variety, or chewing and swallowing difficulties, while physical exercise may be appropriate when oral frailty coexists with reduced muscle strength, mobility, or physical frailty.

Among non-pharmacological interventions, oral exercise has the most consistent evidence for improving specific oral functional outcomes. A systematic review and meta-analysis of 11 randomized controlled trials involving 734 community-dwelling older adults found improvements in tongue–lip motor function, tongue pressure, masticatory function, and maximum occlusal force [50]. Other randomized trials have suggested potential benefits of oral exercise for frailty-related outcomes, while multicomponent interventions combining oral exercise with nutritional and/or physical exercise components have also shown improvements in selected oral and physical function outcomes [50,51,52]. More recent systematic reviews also support non-pharmacological interventions incorporating education, nutrition, and/or physical activity, although the certainty of evidence remains generally low to very low because of heterogeneity in intervention dose, follow-up, and outcome measures [15,53].

A practical management pathway therefore comprises four stages: (1) brief screening with an instrument such as the OF-5; (2) multidimensional assessment of oral, nutritional, physical, and relevant systemic deficits; (3) targeted intervention according to individual needs; and (4) periodic reassessment of oral and systemic function. Mild cases may initially be managed with oral hygiene education, dietary advice, and home-based oral exercises, whereas more substantial dental, swallowing, nutritional, or physical impairments may require professional treatment and multidisciplinary rehabilitation (Table 4).

No universally accepted intervention protocol, optimal exercise dose, or standardized follow-up interval has yet been established. Current evidence therefore supports an individualized, domain-based approach rather than a single standardized treatment regimen. Future randomized trials should determine whether screening followed by targeted intervention can prevent progression from oral frailty to oral hypofunction, physical frailty, sarcopenia, disability, and other clinically meaningful adverse outcomes. Evidence-based interventions specifically targeting oral frailty remain limited. Therefore, some interventions listed below are based on management of the underlying oral or systemic deficits rather than on trials specifically demonstrating prevention of oral frailty progression.

## 7. Integrated Frailty Management Through Medical–Dental Collaboration

The development of oral frailty in older adults is likely driven by complex interactions among biological, socioeconomic, lifestyle, disease-related, and medication-related factors [17]. Oral frailty, physical frailty, and sarcopenia are closely interconnected and may form a self-reinforcing vicious cycle. Declines in masticatory and swallowing function can reduce dietary intake, leading to malnutrition and subsequent losses of muscle mass and physical activity, thereby accelerating physical frailty. Conversely, physical frailty and sarcopenia may further exacerbate oral dysfunction through reductions in occlusal force, tongue pressure, and oral motor performance. These interrelationships highlight the importance of early identification and timely intervention targeting potentially modifiable risk factors.

A meta-analysis of oral frailty and its associated factors among community-dwelling older adults emphasized the importance of early screening and intervention to reduce the risk of adverse outcomes [54]. In addition, an “oral frailty measures program” mentored by dentists and dental hygienists in clinical settings has been shown to improve certain aspects of oral frailty [13]. Comprehensive oral health management has also been recognized as an important strategy for preventing dysphagia-related complications in older adults with Alzheimer’s disease [14]. These findings support an integrated approach in which oral and systemic vulnerabilities are assessed and managed in parallel.

**Figure 4:**
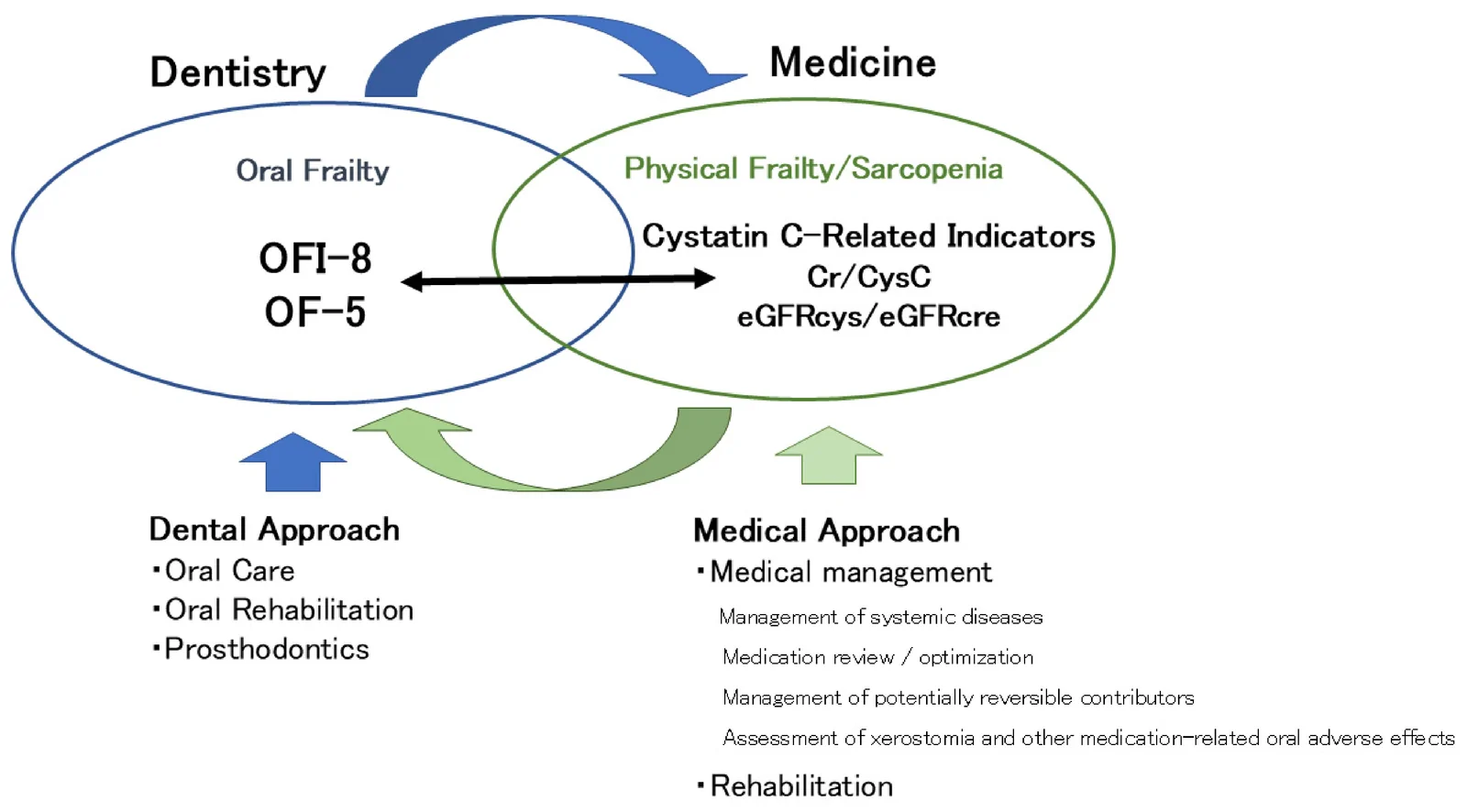
Conceptual framework for the integrated assessment of oral and physical frailty. Questionnaire-based screening tools (OFI-8 and OF-5) are used to assess oral frailty, while cystatin C-related indices provide objective biomarkers reflecting skeletal muscle status and physical frailty. Integrating these complementary approaches may enable earlier detection of frailty and promote multidisciplinary medical–dental collaboration. Reproduced from Reference [39].

Assessment should therefore incorporate both oral and systemic perspectives. Questionnaire-based tools such as the OFI-8 and OF-5 can be used to screen for oral frailty, while cystatin C-related indices, including the Cr/CysC and eGFRcys/eGFRcre ratios, may provide complementary information on systemic vulnerability and physical frailty. Their observed associations with oral frailty suggest potential utility for identifying individuals at increased risk, although these indices should not be regarded as oral frailty-specific biomarkers (Figure 4).

Dental and medical care play complementary roles in this integrated model. Dental care primarily addresses oral hygiene, oral rehabilitation, prosthodontic treatment, and other factors affecting oral function, whereas medical care focuses on systemic conditions associated with physical frailty and sarcopenia through nutritional management, physical rehabilitation, and appropriate medical treatment. Medical assessment should also identify potentially reversible contributors to oral dysfunction, including polypharmacy and medication-related adverse effects.

Medication review is particularly relevant because commonly used medications in older adults may cause dry mouth, altered salivary function, swallowing difficulties, and other oral symptoms. When appropriate, potentially unnecessary medications may be discontinued, doses adjusted, or alternative agents considered in collaboration with the prescribing physician, while ensuring that essential treatments are not compromised. Importantly, no disease-modifying medication has been established for the treatment of oral frailty itself. Thus, medical intervention should be regarded as a complementary component of oral frailty management, primarily addressing systemic diseases and medication-related factors rather than providing pharmacological treatment specifically for oral frailty.

An integrated clinical model combining oral and physical frailty assessment with coordinated medical–dental intervention is therefore an important future goal. Such a model may facilitate early identification of multidimensional vulnerability, individualized intervention, and prevention of progression from oral frailty to oral hypofunction, physical frailty, sarcopenia, and related adverse outcomes.

## 8. Conclusions

Oral frailty represents an early and potentially modifiable state of multidimensional oral vulnerability in older adults and is closely associated with physical, nutritional, and systemic vulnerability. Current evidence supports the use of brief screening tools such as the OF-5 and OFI-8 to identify individuals who may benefit from further assessment, while emphasizing that oral frailty should be distinguished from oral hypofunction, which represents a complementary framework for characterizing established oral functional impairment.

The emerging evidence also suggests that oral frailty should be considered within a broader systemic context. Cystatin C- and creatinine-related indices may provide complementary information on systemic vulnerability, but they should not be regarded as oral frailty-specific biomarkers. Following screening, multidimensional assessment of oral, nutritional, and physical function may help guide individualized interventions, including dental care, oral functional training, nutritional support, physical exercise, and appropriate medical assessment and medication review.

Despite increasing recognition of oral frailty, important uncertainties remain regarding optimal screening instruments, cutoff values, generalizability across populations, biomarker utility, and intervention effectiveness. Future prospective and interventional studies should determine whether systematic identification and targeted management of oral frailty can prevent its progression to oral hypofunction, physical frailty, sarcopenia, disability, and other adverse health outcomes.

## Figures and Tables

**Table 1:** Diagnostic criteria of clinical signs/symptoms of oral hypofunction. Revised from Reference [22].

Clinical Signs	Assessment	Diagnostic Criteria
Poor oral hygiene	Tongue coating	The total number of microorganisms (CFU/mL) is 10^6.5^ or more.
Oral dryness	Oral moisture/salivary flow	The measured value obtained by a recommended moisture checker is less than 27.0.
Reduced occlusal force	Occlusal force measurement	The occlusal force is less than 200 N.
Decreased tongue–lip motor function	Oral diadochokinesis	The number of repetitions of any of /pa/, /ta/, or /ka/ per second is less than 6.
Decreased tongue pressure	Maximum tongue pressure	The maximum tongue pressure is less than 30 kPa.
Decreased masticatory function	Masticatory performance	The glucose concentration obtained by chewing gelatin gummies is less than 100 mg/dL.
Deterioration of swallowing function	Swallowing assessment	The total score of EAT-10 is 3 or higher.

Diagnosis: ≥3 impaired domains.

**Table 2:** Questionnaire enabling simultaneous assessment of OFI-8 and OF-5.

Questions	OFI-8		OF-5	
	Yes	No	Yes	No
***Difficulty in chewing*** Do you have any difficulties eating tough foods compared with 6 months ago?	2	0	1	0
***Difficulty in swallowing*** Have you choked on your tea or soup recently?	2	0	1	0
***Tooth Loss*** Do you use dentures?	2	0		
***Dry mouth*** Do you often experience having a dry mouth?	1	0	1	0
***Social participation*** Do you go out less frequently than you did last year?	1	0		
***Masticatory function*** Can you eat hard foods like squid jerky or pickled radish?	0	1		
***Oral hygiene-related behaviors*** How many times do you brush your teeth in a day? (≥2 times/day)	0	1		
***Oral hygiene-related behaviors*** Do you visit a dental clinic at least annually?	0	1		
***Fewer teeth*** How many of your natural teeth are left? (≥20 natural teeth)			0	1
***Low articulatory oral motor skills*** Have you had difficulty with clear pronunciation recently?			1	0
**Total score**				

Scores are assigned according to the response indicated in the original instruments. **Cut-off Criteria: Criteria:** (1) **OFI-8**: **0–2 points**—Low risk; **3 points**—At risk; **≥4 points**—High risk of oral frailty; (2) **OF-5**: **0–1 point**—Oral frailty negative; **≥2 points**—Oral frailty positive.

**Table 3:** Cystatin C- and creatinine-related biomarkers and their potential relevance to oral frailty.

Biomarker	What it Primarily Reflects	Potential Relevance to Muscle Function	Reported Relationship with Oral Frailty/Oral Function	Interpretation
**Cr/CysC**	Relative creatinine production compared with cystatin C	Lower values may indicate reduced muscle mass or muscle-related vulnerability	Lower in individuals with higher OFI-8-defined oral frailty risk	Promising marker of systemic muscle vulnerability
**eGFRcys**	Kidney function estimated using cystatin C	Relatively less dependent on muscle mass than eGFRcre	Lower values associated with oral frailty in cross-sectional studies	Useful primarily as a renal function marker
**eGFRcre**	Kidney function estimated using creatinine	Strongly influenced by muscle mass	Associations with oral frailty appear less informative when considered alone	Useful as a reference renal function estimate
**eGFRcys/eGFRcre**	Discrepancy between cystatin C- and creatinine-based eGFR	Lower values may indicate low muscle mass or systemic vulnerability	Lower values associated with higher OFI-8-defined oral frailty risk	Promising integrative marker linking renal and muscle vulnerability

Cr, creatinine; CysC, cystatin C; eGFRcre, creatinine-based estimated glomerular filtration rate; eGFRcys, cystatin C-based estimated glomerular filtration rate. Lower Cr/CysC and eGFRcys/eGFRcre values generally indicate greater muscle-related or systemic vulnerability, although these indices are not specific biomarkers of oral frailty.

**Table 4:** Specific Interventions After Oral Frailty Screening.

Problem Identified After Screening	Main Intervention
Tooth loss, denture-related problems, or chewing difficulties	Dental treatment, prosthodontic treatment, and management of chewing function
Poor oral hygiene	Professional oral hygiene care/oral hygiene instruction
Dry mouth (xerostomia)	Assessment of underlying causes and management of oral moisture and salivary function
Reduced tongue and lip motor function	Oral exercises/assessment using oral diadochokinesis (ODK), as appropriate
Reduced tongue pressure	Tongue-resistance exercises
Impaired swallowing function	Swallowing assessment/swallowing exercises
Weight loss or malnutrition	Nutritional assessment and dietary counseling
Reduced muscle strength or physical function	Resistance exercise/physical exercise
Problems across multiple domains	Multidisciplinary intervention
After intervention	Reassessment of oral, nutritional, and physical function

## Data Availability

Data supporting the findings of this study are available from the corresponding author upon reasonable request. However, the data are not publicly available due to privacy and ethical restrictions.
